# Repurposing alternative splicing events as potential targets for the design of diagnostic and therapeutic tools in PCa

**DOI:** 10.3389/fonc.2025.1520985

**Published:** 2025-03-21

**Authors:** Nancy Martínez-Montiel, José de Jesús Vite-Arciniega, Nora Hilda Rosas-Murrieta, Rebeca D. Martínez-Contreras

**Affiliations:** ^1^ Laboratorio de Ecología Molecular Microbiana, Centro de Investigaciones en Ciencias Microbiológicas, Instituto de Ciencias, Benemérita Universidad Autónoma de Puebla, Puebla, Mexico; ^2^ Laboratorio de Bioquímica y Biología Molecular, Centro de Química, Instituto de Ciencias, Benemérita Universidad Autónoma de Puebla, Puebla, Mexico

**Keywords:** splicing, prostate cancer, diagnosis, treatment, RNA

## Abstract

Alternative splicing is a key mechanism responsible for protein diversity in eukaryotes. Even when the relevance of this process was initially overlooked, it is now clear that splicing decisions have a strong impact on the physiology of organisms. Moreover, aberrant splicing products have been clearly related to different diseases, including cancer. Deregulation of splicing factors or mutations at the immature mRNA level could be responsible of generating these aberrant products that are involved in cell biology processes, including migration, angiogenesis, differentiation, cell cycle, DNA repair and so on. For this reason, alternative splicing is now considered a hallmark of cancer. Prostate cancer is one of the most frequently diagnosed types of cancer and some of the leading global cause of cancer death men. Prostate cancer shows an important incidence in the developing world, while the mortality rate is growing because of limited medical infrastructure and awareness. Here, we present some of the key alternative splicing events related to prostate cancer and even when the exact role of these isoforms in the development of the disease has not been fully understood, we believe that the correction of these aberrant splicing events represents an attractive target for the design of innovative diagnostic and therapeutic tools.

## 1 Introduction

Prostate cancer (PCa) is the second most common cancer diagnosed in men (after skin cancer) and the second most common cause of cancer death in men (after lung cancer). Advances in screening, diagnosis, and treatment of PCa have been improving outcomes for thousands of men. In the US, approximately 1 in 44 men will die from PCa in 2024, with an estimated total of 35,250 men, with a 5-year relative survival rate of 97% (1).

Most PCas are found early and are asymptomatic. If symptoms are present, patients may experience difficulty urinating, blood in urine or semen, back pain, or erectile dysfunction.

Screening for PCa is conducted with a PSA test. Although PSA screening can result in early detection of PCa, it is not completely reliable due to the possibility to generate false-positive results and overdiagnosis (2). Due to this possibility, routine PCa screening is not recommended for men at average risk but most organizations endorse shared decision-making to educate men about the pros and cons of PSA screening. In most countries including the US, the course of treatment is not always clear, and there is much debate over which strategies are most effective for PCa in the long term. This landscape urge the necessity to establish a clear correlation between the molecular characteristics and the cellular behavior of the tumor, in order to develop accurate diagnostic tools and effective therapeutic management.

## 2 Current tools for PCa diagnosis

PCa diagnosis requires a combined approach including magnetic resonance imaging (MRI), computed tomography (CT), and positron emission tomography (PET). Additional noninvasive procedures for PCa include serum prostate-specific antigen (PSA) levels and digital rectal examination. Finally, to determine the size and histological grade of the tumor, transrectal and transperineal biopsy, as well as transurethral resection of the prostate, may be performed (3).

In order to properly determine the stage of PCa, the following critical characteristics should be addressed: tumor size and spread to neighboring organs; spread of tumor to lymph nodes; metastasis; histologic grade of the tumor, based on the Gleason score; and PSA levels at the time of diagnosis (4).

Most therapies for early stages of PCa involve either surgically removing the tumor or active surveillance. Because many PCas require testosterone to grow, hormonal therapies aimed at reducing the amount of testosterone are frequently used in addition to medical procedures. Castration-resistant PCa (CRPC) refers to tumors that continue to grow in the absence of testosterone (5). Chemotherapy, immunotherapy, and radio-ligand targeted therapy can be used for CRPC or aggressive and recurrent disease.

Chemotherapeutic agents for PCa include: Cabazitaxel, Carboplatin, Docetaxel and Mitoxantrone hydrochloride. Pembrolizumab and sipuleucel-T are immunotherapy agents used for treating PCa (6).

Targeted therapy can be useful for PCa when the patient has specific mutations or the tumor expresses unique molecular characteristics. The following targeted therapies are applied for PCa: Lutetium lu 177 vipivotide tetraxetan; Olaparib; Radium 223 dichloride and Rucaparib camsylate (6).

Finally, hormonal therapy could be applied for PCa patients in order to reduce testosterone, which is required for tumor growth. The following therapies that work on a patient’s hormonal system are approved for use in PCa: Abiraterone acetate; Apalutamide; Bicalutamide; Darolutamide; Degarelix; Enzalutamide; Flutamide; Goserelin acetate; Leuprolide acetate; Nilutamide; and Relugolix (6).

Even when all these therapeutic tools could be applied in combination to reduce or revert the advance of PCa, most of them are recommended for treatment in advanced stages. In order to produce novel therapies, researchers continue to investigate additional molecular targets for the treatment of PCa.

## 3 Alternative splicing relevance in cancer

Alternative splicing (AS) is a co-transcriptional mechanism that regulates eukaryotic gene expression that affects over 90% of human genes (7, 8). In this mechanism, different combinations of exons and introns can be identified and removed from the pre-mRNA, allowing multiple mRNA configurations of joined sequences to arise from a single gene, increasing the coding potential of the genome (9).

Malfunctions of alternative splicing events can affect the natural expression of a large number of transcripts, including several factors involved in apoptosis or cell survival, molecular processes intimately associated with cancer evolution (10, 11). In many cases, specific splicing factors or mutated components of the splicing machinery are linked to an anomalous event. Moreover, a switch in specific splicing events occurs in particular types of cancer where the concomitant outcome is the production of non-functional proteins with added, deleted, or altered domains affecting tumorigenesis (12). With all this evidence, several strategies have been developed to regulate alternative splicing, some oriented to improve cancer prognosis, therapeutic and treatment.

Alternatively spliced messenger RNA often produce proteins with distinct or even opposing functions (i.e. Bcl-x produces a pro-apoptotic and an anti-apoptotic versions). It is estimated that as much as 50% of all genetic disorders may be caused by mutations that alter pre-mRNA splicing (13). There is increasing evidence regarding genes involved in different stages of cancer, whose alternative splicing is affected, with consequences in different processes such as cellular invasion and proliferation, resistance to apoptosis and susceptibility to different chemotherapeutic agents. In accordance with the information provided by the Cancer Genome Project of the Wellcome Trust Sanger Institute, 488 human genes possess mutations associated with some type of cancer. More relevant, 63 of them present mutations that somehow affect their alternative splicing. Despite the relevance of splicing to diseases, few approaches exist to control this mechanism with therapeutic purposes (14).

## 4 Alternative splicing events related to PCa

Even when more evidence concerning the relevance of alternative splicing in cancer is unraveled every day, further studies are needed in order to analyze the molecular mechanisms regulating aberrant splicing events and the cellular pathways altered by these splicing decisions. Here, we present some of the key splicing events associated to PCa development. Each case is presented separately and the key information is summarized in **Table 1**.

**Table 1:** Splicing events relevant to prostate cancer.

Gene official symbol/Official full name	Splicing events, mutations	Functional impacts	Potential as therapeutic targets	Reference
AR androgen receptor	Aberrant alternative splicing	Synthesis of two truncated isoforms of the constitutively active receptor, called AR-V7 and AR-V9, which lack the LBD domain (exons 5 to 8). The isoforms correlate with cell proliferation, resistance and protection against hormonal treatments.		(15) (16) (17)
	Dysregulation and overexpression of the SRSF1 splicing factor	Synthesis of aberrant AR-V7 and AR-V9 isoforms correlates with SRSF1 overexpression.		(18)
BCL2L1 BCL2 like 1 or *BCL-X* gene	Isoforms BCL-XL and BCL-XS from two alternative 5’ splice sites in the exon 2. Regulation of isoforms expression by the cell cycle and activation of phosphatases.	Prostate cell lines expressing BCL XL (phosphorylation-activated protein) exhibit a high resistance to apoptosis, as well as resistance to various treatments, from chemotherapy to radiotherapy.	Phosphorylase inhibitor drugs.	(21) (22)
CCND1 cyclin D1	SNP G/A870 at the end of exon 4 and beginning of intron 4.	The isoform called cyclin D1b retains a portion of intron 4 that includes a premature stop codon. Cyclin D1b is in the nucleus and correlates with decreased activation of the androgen receptor.		(23)
CD44 CD44 molecule (IN blood group)	Dysregulation of alternative splicing with diverse isoforms at different stages of the disease.	There is a downregulation of CD44 expression in metastatic stages of prostate cancer, while migration is decreased.	CD44 and CD44v6 could be markers for finding prostate metastatic cells in liquid biopsies.	(24)
		CD44v6 is expressed in the epithelial to mesenchymal transition.		(25)
KLK3 kallikrein related peptidase 3 (Prostate Specific Antigen)	Highly complex alternative splicing events, SNPs.	Overexpression of KLK3 degrades the extracellular matrix and thus expands cancer tissue, and correlates with increased angiogenesis.	New and improved diagnostic and therapeutic tools based on	(26)
	Highly complex alternative splicing events, SNPs.		KLK3 variants to assess disease progression.	(27)
FOXA1 forkhead box A1	High rate of mutagenesis in any endoderm-derived tissue, such as the prostate gland.	The effect on splicing regulation is not yet fully understood.		(28) (29)
IGF1 insulin like growth factor 1	Alternative splicing, three isoforms called IGF-IEa, IGF-IEb and IGF-IEc.	In PCa epithelial cells, the IGF-IEc isoform is highly expressed with an increase in proliferation mediated by activation of the IGF-1R receptor. Association between increased circulating levels of IGF-1 and the risk of developing solid malignancies.	IGF-1 and IGF-IEc could act as circulating biomarkers.	(30) (31)
KLF6 KLF transcription factor 6	IVSΔA allele generate the alternative isoforms not functional: KLF6- V1, KLF6-V2 and KLF6-V3. The IVSΔA allele has a G/A polymorphism that generates a new splice site near the boundary between the first intron and the second exon called the IVS1-27 point.	The isoforms act as antagonists of the functional KLF6 isoform, cancers tend to be aggressive and metastasis.	IVSΔA allele related to PCa in men, in the American population.	(32) (33)
ERG/TMPRSS2 ERG ETS transcription factor ERG TMPRSS2 transmembrane serine protease 2	Gene fusion with the androgen-driven promoter of the TMPRSS2 gene through chromosomal translocation or by interstitial deletion of the intergenic region between TMPRSS2 and ERG.	ERG/TMPRSS2 deletion is present in approximately 50% of PCas.	Modify the splicing events of ERG gene.	(34)
	Up to 30 alternative transcripts of the ERG gene.	ERG is phosphorylated by ERK kinase, triggering the posttranslational activity that drives cell proliferation.		(35) (36)
NF-YA nuclear transcription factor Y subunit alpha	Alternative splicing produces the NF-YAL and NF-YAS isoforms.	Increased NF-YAS induces cancer cell proliferation, while overexpression of NF-YAL increases cell mobility.	New molecular strategy for risk assessment of PCa patients.	(37, 38)
VEGFA vascular endothelial growth factor A	Alternative slicing produces angiogenic and anti-angiogenic isoforms.	In PCa cell lines, the ratio between these pro-angiogenic and anti-angiogenic isoforms is affected, with a tendency to increase for proangiogenic isoforms.		(39) (40)
	Dysregulation of the SRSF1 factor.	Dysregulation of the SRSF1 factor increases the expression of VEGFA121 (angiogenic) and decreases the isoform with better anti-angiogenic potential.		(41)

### 4.1 The androgen receptor

Alternative splicing of the androgen receptor is one of the most studied events for PCa. This nuclear receptor for male sex hormones is a multidomain protein that possess the ability to dimerize when binding to an androgen molecule and the activation of this route starts different mechanisms of cell development and proliferation (42).

**Figure 1:**
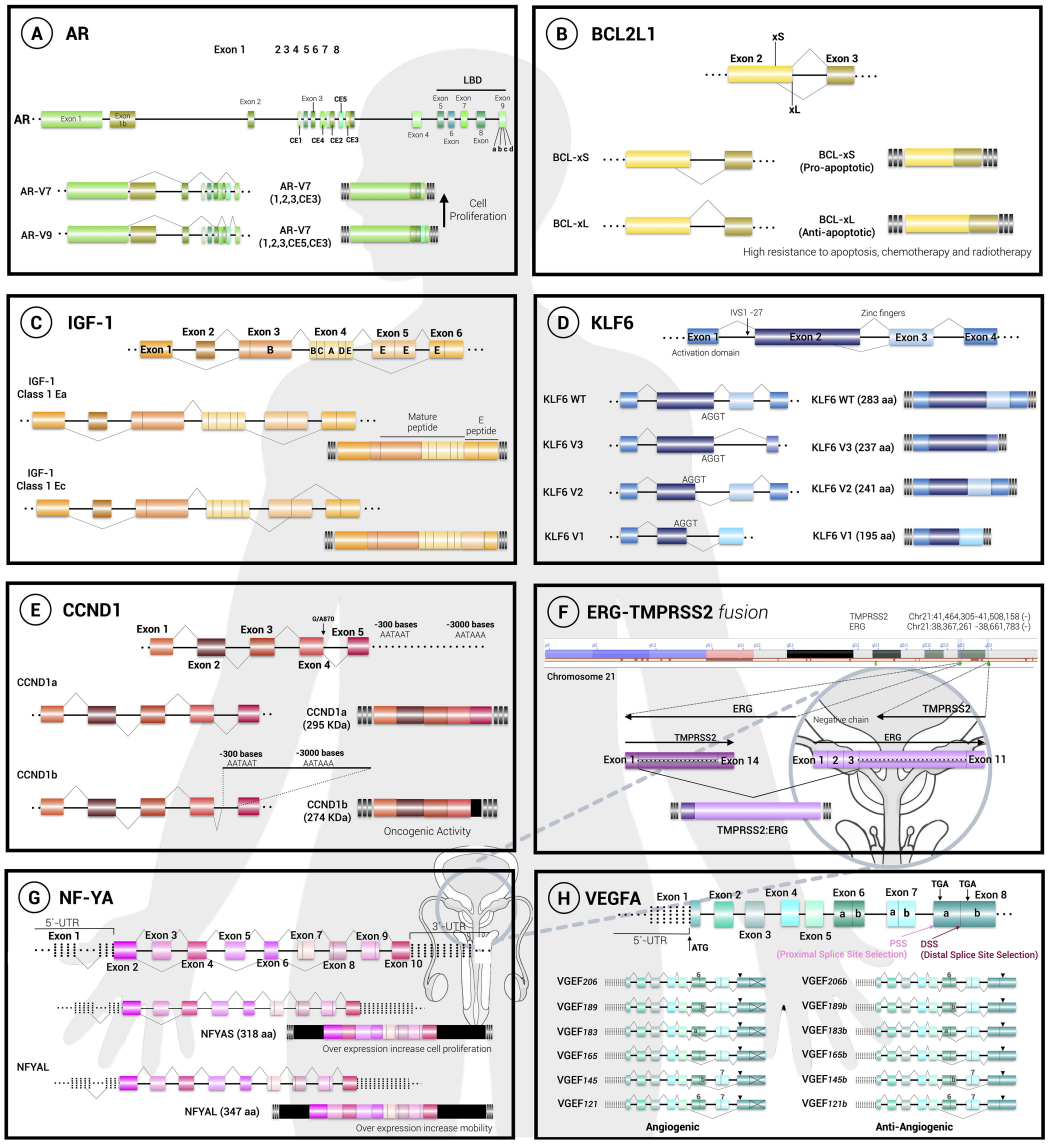
Alternative splicing decisions regulate cell fate. The general structure and splicing choices are depicted for 8 pre-mRNAs, as follows: **(A)** the androgen receptor (AR), **(B)** the BCL-X gene (BCL2L1), **(C)** the insuline-like growth factor (IGF), **(D)** the krüppel-like transcription factor 6 (KLF6), **(E)** D1 (CCND1), **(F)** the fusion between the ETS-related gene and the transmembrane serine protease 2 gene (ERG/ TMPRSS2), **(G)** the nuclear factor Y (NF-YA) and **(H)** the vascular endothelial growth factor A (VEGFA). The cellular effect of the isoforms generated is mentioned. Exons are represented with boxes while lines correspond to introns.

The gene coding for the AR consists of 8 exons, where exons 5 to 8 encode the ligand-binding domain (LBD). Interestingly, in prostatic cancer cells the expression of this gene is modified and an aberrant alternative splicing occurs (**Figure 1A**), generating two truncated isoforms of the receptor, named AR-V7 and AR-V9 (43). These isoforms completely lack the LBD domain and therefore they are not able to bind to androgens, but they are constitutively active, so that they cannot be negatively regulated and will be constantly generating proliferation, conferring resistance and protection against hormonal treatments (15–17).

The expression of these aberrant isoforms derived from a deregulated alternative splicing event is not directly associated with a mutation in the gene for the androgen receptor, however it has been pointed out that the level of expression of these isoforms is related to an increase in the number of copies within the locus containing the gene in the cell. Moreover, it has been depicted that a deregulation and overexpression of the SRSF1 splicing factor correlates with the appearance of aberrant isoforms (18).

### 4.2 BCL2L1

The *BCL-X* gene (or *BCL2L1*) regulates apoptosis and therefore has an extremely important role in cancer (19, 20). This gene is constituted by 3 exons with the ability to generate 2 isoforms due to the selection of two alternative 5’ splice sites in exon 2, resulting in isoforms BCL-X_L_ (B-Cell Lymphoma xtra large) and BCL-X_S_ (B-Cell Lymphoma xtra small). Both isoforms have opposite functions where BCL-X_S_ is proapoptotic being part of the core apoptosome, while on the contrary BCL-X_L_ is antiapoptotic by antagonizing proapoptotic functions of other molecules belonging to the BCL-2 family (**Figure 1B**).

The regulation of the expression of these isoforms is determined by the regulation of the cell cycle and with this, the activation of different phosphatases. At the level of splicing regulation, it is known that the expression of BCL-X_S_ is determined by hnRNP H and F, SAM68 and RBM25 (44–46). On the other hand, the expression of BCL-X_L_ is regulated by SR proteins such as SRSF1, SRSF9 and SAP155 (45–48) that must be phosphorylated and this modification usually occurs during the initiation of cell replication (22). The disruption of the delicate balance between BCL-X_L_ and BCL-X_S_ isoforms has not been related to a specific mutation; however, it is believed that it has to do with the appearance of epigenetic changes that affect the cell cycle, but further evidence is needed to fully demonstrate this observation.

In the context of PCa, it has been determined that prostatic cell lines expressing BCL-X_L_ show a fairly high resistance to apoptosis generating resistance to various treatments from chemotherapy to radiotherapy (21). As an alternative, phosphorylase inhibitor drugs have been implemented for cancer treatment, although this could generate negative effects in other healthy tissues.

### 4.3 CCND1

The *CCND1* gene consists of 5 exons and encodes the cyclin D1 protein which is a proto-oncogene given its ability to activate cyclin-dependent kinases (CDK) 4 and 6 forming a complex that phosphorylates and thus activates molecules that allow cell cycle progression from G1 to S phase promoting cell proliferation. Moreover, cyclin D1 sequesters inhibitors of CDK kinases such as p27.

Usually, the mRNA resulting from the *CCND1* gene does not undergo alternative splicing; however, it has been seen that in multiple cancers and tumors there are translocations and deletions that promote the activation of this gene (49). In PCa, it has been determined that a single nucleotide polymorphism for this gene is common, this allele called *G/A870* consists of the change of a guanine by an adenine in the vicinity of the end of exon 4 and beginning of intron 4. This mutation causes a decrease in the recognition of the usual splice sites and favors the formation of a transcript that retains a fraction of intron 4 that includes a premature stop codon, generating the isoform called cyclin D1b (**Figure 1E**). This isoform always resides in the nucleus, unlike the normal isoform that shuttles between the nucleus and the cytoplasm according to the signals in the cell. The expression of this isoform also correlates with a decrease in the activation of the androgen receptor in PCa (23).

### 4.4 CD44 and *KLK3*

CD44 is an adhesion glycoprotein expressed in the epithelial cells of prostate tissue. The *CD44* gene consists of 20 exons, where intermediate exons v2 to v10 are mutually exclusive and could be included due to alternative splicing decisions. It has been reported that during PCa a deregulation of this alternative splicing selection occurs, generating various isoforms at different stages of the disease. However, it has been observed that in metastatic stages of PCa there is a negative regulation in the expression of this glycoprotein while migration is decreased (24). One report proposes that CD44v6 is expressed in epithelial to mesenchymal transition (25). Considering this evidence, CD44 and CD44v6 could be an interesting marker to find prostatic metastatic cells in liquid biopsies.

The *KLK3* gene encodes the protein peptidase related to kallikrein 3 (KLK3), better known as the prostate specific antigen (PSA). KLK3 functions as a protease and it plays an extremely important role during the development of PCa, where it is overexpressed in order to degrade the extracellular matrix and thus expand the cancerous tissue (26). Moreover, an increase in angiogenesis also correlates with the overexpression of KLK3 in the context of PCa (27).

KLK3 consists of 5 exons and 8 isoforms of the KLK3 protein are known. Even when different attempts have been made in order to relate the different isoforms of KLK3 with malignancy and progression of PCa, no irrefutable evidence has been found yet and it becomes interesting to explore alternative splicing regulation for this PCa biomarker (26).

Both the *CD44* and *KLK3* genes are important in the development of PCa disease and although they are well studied, alternative splicing events are highly complex, and not only have polymorphisms been reported that modify the dynamics of splicing, It has also been seen that this modification can be orchestrated by epigenetic mutations and therefore are not very useful as a therapeutic target. Interestingly, there was a sharp decline in the overall incidence of PCa from 2007 to 2014 in the US, which correlated with a reduction in prostate specific antigen (PSA) screening as a result of changes to US Preventive Services Task Force recommendations. Variations in PCa incidence rates mainly reflect the use of PSA screening, which typically detects localized disease. In 2023, it was estimated that 55% of American men with metastatic PCa were initially diagnosed with localized or regional disease, reflecting the importance of PSA detection. Even when the full impact of splicing decisions for CD44 and KLK3 in the onset an evolution of PCa remains elusive, it results interesting to analyze the expression of splicing variants during the progression of the disease in order to develop new and improved diagnostic and therapeutic tools.

### 4.5 FOXA1

FOXA1 is a transcriptional factor responsible for the modulation of key splicing factors such as hnRNPK and SRSF1 (50). Although several mutations have been detected in the FOXA1 gene, the effect of these mutations on the expression of genes regulated by splicing factors including SRSF1 (like AR or VEGFA) has not been fully understood yet. The *FOXA1* gene has been found to have a very high mutagenesis rate, which is reflected in the ability to generate tumors in basically any endoderm-derived tissue, including the prostate gland (28).

The relationship between FOXA1 and splicing regulation in the context of PCa is still not fully understood (29), but we believe that the analysis of this cellular cascade in the following years could mean a significant advance for the development of diagnostic targets.

### 4.6 IGF-I

Insulin-like growth factor I (IGF-I), also called somatomedin C, is essential in developmental and proliferative functions in many tissues, besides maintaining homeostasis of several hormones including insulin. This factor is secreted mainly by the liver and some other tissues such as the prostate in a paracrine manner.

The gene encoding this factor (*IGF-I)* consists of 6 exons that generate 3 different isoforms: IGF-IEa, IGF-IEb and IGF-IEc (30) and the IGF-IEc isoform is highly expressed during PCa and this has been directly related to an increase in proliferation mediated by the activation of the IGF-1R receptor in PCa epithelial cells (**Figure 1C**). Interestingly, several studies have shown an association between increased levels of circulating IGF-1 and the risk of developing solid malignancies, including PCa (31). The IGF-IEc isoform is overexpressed in human PCa tissues and in human PC-3 and LNCaP cells. This preferential IGF-IEc mRNA expression generates the MGF E peptide that possesses mitogenic activity through mechanisms independent of IGF-1R, IR, and hybrid IGF-1R/IR (51). Altogether, these observations suggest that IGF-1 and IGF-IEc could act as circulating biomarkers for PCa diagnosis.

There are also reports of mutations that favor the formation of other IGF-I isoforms, although they have not yet been related to PCa.

### 4.7 KLF6

The *KLF6* gene (krüppel-like transcription factor 6), consisting of 3 DNA-binding zinc domains, regulates the cell cycle by inducing the expression of the cell cycle inhibitor p21 and preventing replication as its main function; however, KLF6 is a factor that can also be one of several modulators of cell differentiation, apoptosis and development and is therefore considered a tumor suppressor.

Usually, KLF6 is expressed without undergoing alternative splicing (wtKLF6 isoform). However, individuals possessing the IVSΔA allele generate 3 alternative isoforms: KLF6-V1, KLF6-V2 and KLF6-V3. These isoforms lack one (KLF6-V2) or 2 zinc domains (KLF6-V1 and KLF6-V3) and therefore are not functional (**Figure 1D**). In addition, alternative variants can also act as antagonists of the functional isoform wtKLF6 in such a way that it behaves as a dominant allele and therefore cancers related to this isoform tend to be aggressive and generate metastasis. The IVSΔA allele possesses a unique G/A polymorphism that generates a new splice site near the boundary between the first intron and the second exon (called IVS1 -27 point) that is recognized by the SRp40 protein and promotes the expression of isoforms lacking zinc domains (32).

The presence of the IVSΔA allele has been studied only in the American population and it has been determined that it is related to lung and PCa in men (33).

### 4.8 ERG/TMPRSS2

The *ERG* gene (ETS-related gene) is a member of the E-26 transformation-specific (ETS) family of transcription factors. The ETS transcription factors have a pivotal role in development and cell differentiation with roles in different cell types, where it regulates embryogenesis, vasculogenesis, angiogenesis, haematopoiesis and neuronal development. This transcription factor regulates the expression of genes involved in the regulation of cellular architecture, cell migration, invasion and cell permeability.

There are several descriptions of *ERG*’s gene and exon/intron structure. The *ERG* locus is approximately 300 kb long and includes at least 12 exons producing up to 30 alternative *ERG* transcripts that are expressed and encoding at least 15 protein variants (**Figure 1F**).

Prostate epithelia do not normally express *ERG*, but it has been reported that *ERG* is overexpressed in a high proportion of prostate carcinomas as a result of a gene fusion with the androgen-driven promoter of the *TMPRSS2* gene. This fusion is caused by chromosomal translocation or by interstitial deletion of the intergenic region between *TMPRSS2* and *ERG* (34). Deletions may occur because of fragile sites and breakpoints found in intron 2 of *ERG* and in introns 1 and 2 of *TMPRSS2* that resemble Alu repeat elements. These elements are involved in gene expression at different levels, including alternative splicing. This aberrant ERG represses a number of prostate epithelium-specific genes, including *KLK3*, suggesting that ERG promotes the de-differentiaton of prostate epithelium (52).

Another alternative splicing decision relevant for ERG expression in prostate cancer involves cassette exons 7 and 7b. It has been reported that when exon 7b is included, ERG is phosphorylated by ERK kinase, triggering the posttranslational activity that drives cell proliferation (35).

It has been seen that this *ERG/TMPRSS2* deletion is present in approximately 50% of PCas and it has been demonstrated that inhibiting the insertion of exon 7b decreases cell proliferation and migration. Due to the complexity of this gene, no trans acting splicing regulatory factors have been described yet but the development of tools designed to modify these splicing events are attractive (36).

### 4.9 Nuclear factor Y

The transcription factor NF-Y supports cell proliferation by activating the transcription of various molecules that allow transformation and proliferation. This factor comprises 3 subunits: NF-YA, NF-YB and NF-YC. The gene coding for the NF-YA subunit consists of 9 exons where the first exon corresponds to a non-coding sequence and the third exon undergoes alternative splicing that encodes 28 amino acids (**Figure 1G**), producing the NF-YA_L_ and NF-YA_S_ isoforms, respectively (37). Although the dynamics that deregulates the expression of these isoforms is not fully known, it has been seen that other epithelial cancers such as lung adenocarcinoma overexpress the long isoform NF-YA_L_ which has been related to cell migration, while the short isoform NF-YA_S_ is related to endometrial cancer tumors. In PCa, it has been observed that the increase of NF-YA_S_ induces cancer cell proliferation, while NF-YA_l_ overexpression increases cell motility. We believe that evaluation of NF-YA splicing may represent a new molecular strategy for risk assessment of PCa patients (38).

### 4.10 VEGFA

The vascular endothelial growth factor or VEGFA promotes angiogenesis and the gene that encodes this factor can generate different angiogenic isoforms that are generally referred to as “VEGFAxxx” where xxx refers to the number of amino acids present in the protein. According to this, each isoform has a different half-life and activates various signaling pathways (**Figure 1H**).

The angiogenic isoforms VEGFA121, VEGFA145, VEGFA148, VEGFA165 and VEGFA206 are normally expressed in cells but they show a certain ratio with the anti-angiogenic isoforms (VEGFA121b, VEGFA145b, VEGFA148b, VEGFA165b, VEGFA206b), so the regulation of their expression is essential for the correct functioning of the tissue (39). However, in PCa cell lines it has been found that the ratio between these pro-angiogenic and anti-angiogenic isoforms is affected and expression tends to be enhanced for the pro-angiogenic isoforms (40). As in the case of the androgen receptor (AR), this change in isoform regulation is not related to a mutation in the VEGFA gene so far, but it is the dysregulation of the SRSF1 factor that mainly increases the expression of VEGFA121 and decreases the isoform with the best anti-angiogenic potential (41).

## 5 Concluding remarks

Alternative splicing has a key role in the physiology of prostate cancer. In the near future, it will be interesting to direct our efforts to connect the molecular evidence with the cellular behavior that prevails in the context of prostate cancer. This landscape could help us to properly develop new tools to innovate diagnostic and therapeutic tools in order to improve the outcome for cancer patients. The possibility to monitor splicing events in liquid biopsies would be a non-invasive tool for cancer diagnosis, while RNA molecules designed with therapeutic applications is now a reality. With all this in mind, we hope that alternative splicing modulation will become an alternative for prostate cancer patients.
